# Circadian Patterns in Postvoid Residual and Voided Percentage among Older Women with Urinary Incontinence

**DOI:** 10.3390/jcm9040922

**Published:** 2020-03-27

**Authors:** Veerle Decalf, Thomas F. Monaghan, Marie-Astrid Denys, Mirko Petrovic, Ronny Pieters, Jeffrey P. Weiss, Karel Everaert

**Affiliations:** 1 Faculty of Medicine and Health Sciences, Department of Human Structure and Repair, Ghent University, 9000 Ghent, Belgium; marie-astrid.denys@ugent.be (M.-A.D.); karel.everaert@ugent.be (K.E.); 2 Department of Urology, State University of New York Downstate Health Sciences University, Brooklyn, NY 11203, USA; thomas.monaghan@downstate.edu (T.F.M.); jeffrey.weiss@downstate.edu (J.P.W.); 3 Department of Geriatrics, Ghent University Hospital, 9000 Ghent, Belgium; mirko.petrovic@uzgent.be; 4 Faculty of Medicine and Health Sciences, Department of Internal Medicine and Pediatrics, Ghent University, 9000 Ghent, Belgium; 5 Department of Urology, Ghent University Hospital, 9000 Ghent, Belgium; ronny.pieters@uzgent.be

**Keywords:** age, circadian, diagnostic, female, imaging, pathophysiology

## Abstract

Background: Women with urinary incontinence incur an increased risk of elevated postvoid residual (PVR) volume and impaired voiding efficiency (i.e., voided percentage (Void%)), but the clinical significance of these parameters remains poorly described. Further characterization of PVR and voiding efficiency may thus be useful in refining the evaluation and management of urinary incontinence. This study aims to explore possible circadian variations in PVR and Void% in older women with stress (SUI), urge (UUI) and mixed urinary incontinence (MUI). Methods: A single center prospective study which enrolled a convenience sample of 90 older women who consulted a tertiary referral hospital for urinary incontinence. Participants underwent an extensive medical interview and were hospitalized to complete a 24-h frequency-volume chart (FVC) with PVR measurement after each void (FVC_PVR_). Results: FVC_PVR_ analysis demonstrated no differences in mean PVR and Void% between patients with SUI, UUI and MUI. Likewise, no daytime or nighttime differences were observed in mean PVR or Void% within or between groups. Conclusions: No evidence of circadian variation in PVR or Void% was observed in older women with SUI, UUI or MUI.

## 1. Introduction

Postvoid residual (PVR) is the volume of urine that remains in the bladder after voluntary micturition [1]. An increased PVR (i.e., incomplete bladder emptying) may be a cause of lower urinary tract symptoms (LUTS) such as urgency, frequency, incontinence and nocturia and, in some cases, may also contribute to upper urinary tract dysfunction [2]. The voided percentage (Void%), defined by the International Continence Society (ICS) as the proportion of bladder content emptied [3], is even more strongly associated with peak flow rate (Qmax) [4], and may thus also be a clinically relevant variable in the evaluation of LUTS mediated by inefficient voiding. 

Although there is no consensus as to what volume constitutes an “elevated” PVR [5], and the specific cutoffs employed may vary across different LUTS, values of 50–100 mL are commonly recognized as the floor threshold for abnormal [6], as PVR values in excess of 100 mL are exceedingly rare in middle-aged and older women [7]. Despite some heterogeneity across study populations, PVR values greater than 100 mL may be relatively more prevalent among women with stress urinary incontinence [8] and urge urinary incontinence [9].

Problematically, across all LUTS, significant intra-individual variability in PVR volumes and Void% generally exists, which hinders their potential diagnostic utility in LUTS management [2]. Recent research of uroflowmetry parameters in women with different subtypes of urinary incontinence indeed demonstrated high intra-individual variability in PVR volumes, but did not account for the potential influence of time-of-day on study results [10], which has been recognized as a potential confounding variable in the association between LUTS severity and PVR [11].

Further characterization of the interplay between time-of-day, PVR and voiding efficiency may thus be useful in refining the evaluation and management of urinary incontinence. Although frequency-volume chart (FVC) data has, to our knowledge, never been recorded with PVR across a 24-h period, previous small-scale research involving geriatric subjects with incontinence reported significantly higher intrasubject PVR values in the early morning compared to the afternoon or evening [11]. Accordingly, we aimed to test the hypothesis that PVR is greater in the nighttime vs. daytime in women with urinary incontinence. Secondly, we aimed to characterize circadian variations within and between subgroups of women with stress (SUI), urge (UUI) and mixed (MUI) urinary incontinence.

## 2. Materials and Methods

### 2.1. Study Design and Participants

This study undertook a post hoc analysis of unpublished data from the Think Dry cross-sectional descriptive study of urinary incontinence in older adults. The study population consisted of a convenience sample who consulted a tertiary referral hospital for urinary incontinence from December 2013 to December 2018. Included were patients aged ≥ 65 years with a chief complaint of urinary incontinence. Exclusion criteria were a positive screen for cognitive impairment on the Mini-Cog [12], symptomatic urinary tract infection and incontinence associated with recurrent infection, pain, hematuria, pelvic irradiation, radical pelvic surgery, suspected fistula or urinary retention. Local ethics committee approval (2013/950) was obtained, the Declaration of Helsinki was followed, and all participants provided written informed consent. 

### 2.2. Study Protocol

All Think Dry participants completed a 24-h frequency-volume (FVC) chart and PVR (FVC_PVR_), a renal function profile (RFP) [13] and urodynamic testing. The data amassed were used by a functional urology specialist (K.E.) to establish specific diagnoses. Participants also completed several validated questionnaires, which were used to characterize their functional status (Belgian-modified Katz Index of Independence in Activities of Daily Living (ADL) [14]), frailty (Tilburg Frailty Indicator (TFI) [15]), fall risk (St. Thomas’s Risk Assessment Tool in Falling Elderly Inpatients (STRATIFY) [16]) and the impact of LUTS on their quality of life (International Consultation on Incontinence modular Questionnaire for LUTS in women (ICIQ-fLUTS) [17]).

For the present analysis, female patients with a diagnosis of urodynamic stress incontinence and/or detrusor overactivity who completed a FVC_PVR_ were included, such that 11 patients were excluded because urinary incontinence was not reproduced during urodynamic testing.

### 2.3. Materials

The Katz Index dichotomizes independence/dependence for six ADLs (bathing, dressing, transferring, toileting, continence and feeding). Scores are summated, such that a score of six reflects fully preserved function, four indicates moderate impairment, and ≤2 indicates severe functional impairment [18].

The TFI is a 15-item questionnaire assessing physical, psychological and social frailty, with increasing scores reflecting increased impairment in these domains. For this instrument, the maximum total score is 15, and patients with a score ≥ 5 may be deemed frail [15].

The STRATIFY instrument consists of five items that evaluate risk factors for falling. Scores range from zero to five, with a score ≥ 2 indicating high fall risk [16]. 

The ICIQ-fLUTS questionnaire provides three subscores for filling (0–16), voiding (0–12) and incontinence symptoms (0–20), wherein higher scores correlate with symptom severity [17]. 

All FVC_PVR_ measurements were obtained by nurses of the inpatient urology department. Nurses recorded the voided volume (VV) (mL), urinary incontinence (UI) weight (g) and PVR (mL) accompanying each micturition, as well as patients’ time to bed with the intention of sleeping and time of awakening. PVR was analyzed noninvasively using a validated portable ultrasound device (BladderScan® BVI 9400, Verathon, Inc., Bothell, WA, USA) within 15 min after each micturition [19]. Patients were permitted to consume fluids and solids ad libitum in an effort to best simulate real-world conditions.

Measurements derived from the FVC_PVR_ were defined in accordance with reports from the Standardization subcommittee of the ICS [1,20]. Namely, global polyuria was defined as a 24-h urine output > 40 mL/kg; and nocturnal polyuria (NP) was defined as a nocturnal urine volume > 33% of the total 24-h urine volume in the absence of global polyuria. Nocturia was defined as at least 2 nocturnal voids, because this is the threshold at which most patients are more likely to report clinically significant nocturia-related bother and experience impaired health-related quality of life [21]. Voided percentage (Void%) was calculated as {(VV/[VV + PVR]) × 100} [3]. Missing values were not estimated or replaced, except in the case of one missing daytimevalue, for which values were replaced with corresponding mean daytime values for that patient (*n* = 9).

Urodynamic studies were conducted according to local protocols, which were guided by current ICS recommendations [22].

### 2.4. Statistical Analysis

Between incontinence subgroups, continuous and categorical variables were compared using the Kruskal-Wallis and Fisher’s exact tests, respectively. A pairwise comparison using the Mann-Whitney *U* and Fisher’s exact tests for continuous and categorical variables, respectively, was used to establish partial order between pairs when significant differences were identified on three-way analysis, with a Bonferroni correction (*p* < 0.017) applied for multiple comparisons. The Wilcoxon Signed-rank test was used to assess intragroup differences in PVR and Void%. Continuous variables are reported as median (confidence interval), and categorical variables are reported as frequency (percent). Data were analyzed using Statistical Package for Social Sciences (Version 24, IBM Corp., Armonk, NY, USA).

## 3. Results

### 3.1. Study Population

A total of 90 patients were included, for which the median age was 76 (72–80) years and parity was 2 (1–3) children (Table 1). Most of the participants (98%) lived in the community, while two (2%) were nursing home residents. The distribution of hysterectomy (*p* = 0.031), depression (*p* = 0.048), neurological lesions (*p* = 0.023) and utilization of selective serotonin reuptake inhibitors (SSRIs) (*p* = 0.032) were recorded differed between groups. History of hysterectomy was more prevalent in patients with SUI compared to UUI and MUI (57% vs. 28% and 27%, *p* = 0.019 and *p* = 0.026, respectively). Patients with UUI were more likely to be affected by depression compared to SUI (25% vs. 4%, *p* = 0.033). Neurological disorders were more prevalent in patients with UUI compared to SUI (31% vs. 4%, *p* = 0.008). SSRI utilization was greater among patients with UUI vs. SUI (28% vs. 4%, *p* = 0.017). No further significant pairwise differences were observed for these parameters.

For questionnaire data, the distribution of functional status (Katz Index) and frailty (TFI) significantly differed between subgroups. (Table 2). A greater proportion of patients with SUI reported a well-preserved ADL function compared to patients with UUI (93% vs. 47%, *p* < 0.001). Consistently, fewer patients with SUI reported severe ADL impairment compared to those with UUI (0% vs. 31%, *p* < 0.001). Frailty on the TFI was more prevalent among the UUI subgroup compared to patients with SUI (85% vs. 52%, *p* = 0.010). No further three-way or pairwise differences were observed in questionnaire analysis.

### 3.2. FVC_PVR_ Characteristics

The FVC_PVR_ characteristics for each subgroup are presented in Table 3. Between subgroups, significant differences were observed in 24-h volume, proportion of patients with global polyuria, mean VV, 24-h maximum VV and total incontinence weight. The 24-h urine output was significantly higher in patients with SUI (2549 mL) vs. UUI (1747 mL) (*p* < 0.001) and in MUI (2173 mL) vs. UUI (*p* = 0.004). Consistently, global polyuria was more prevalent in patients with SUI (29%) vs. UUI (6%) (*p* = 0.016), and in patients with MUI (31%) vs. UUI (*p* = 0.013). The median number of voids in 24 h was higher in MUI (10 voids) vs. SUI (9 voids) (*p* = 0.010) or UUI (8 voids) (*p* = 0.003). Mean and maximum voided volumes were greater in patients with SUI (291 mL, 470 mL) than in UUI (177 mL, 335 mL) patients (*p* < 0.001, *p* < 0.001) and MUI (181 mL, 320 mL) patients (*p* = 0.002, *p* = 0.001). No pairwise differences were observed in incontinence weight after Bonferroni correction.

No circadian differences were observed in mean PVR or Void% between subgroups. Moreover, within groups, no significant differences were observed between daytime and nighttime mean PVR for SUI (22 vs. 20 mL, *p* = 0.764), UUI (17 vs. 16 mL, *p* = 0.905), or MUI (22 vs. 31 mL, *p* = 0.107). Likewise, no significant intragroup difference was observed between daytime and nighttime Void% for SUI (91 vs. 94%, *p* = 0.394), UUI (89 vs. 91%, *p* = 0.617), or MUI (92 vs. 91%, *p* = 0.977). The distribution of mean 24-h and maximum daytime PVR is presented in Table 4.

## 4. Discussion

To our knowledge, this is the first study to utilize the 24-h FVC with PVR volume to characterize the circadian pattern of PVR and Void% in older women with SUI, UUI and MUI. In the present analysis, no significant circadian variations were observed in mean PVR or Void% within subgroups.

Abnormal residual volume is a common phenomenon in the setting of urinary incontinence. Although there is no consensus as to what exactly constitutes an elevated PVR, many experts have proposed values between 50–100 mL as the lower threshold for abnormal residual urine volume [24]. In their analysis of PVR in middle-aged women with urodynamic stress incontinence, Tseng et al. recognized that more than one-third of participants had a PVR > 50 mL, and nearly one in five experienced a PVR > 100 mL [8]. Consistently, in the present analysis, a maximum daytime PVR >50 mL was identified in more than half of all participants, and a maximum daytime PVR > 100 mL was recorded in one-third of all subjects. 

Importantly, however, while more than one-fourth of all participants in the present study demonstrated mean 24-h PVR > 50 mL, only 7% of all patients experienced a PVR > 100 mL. Thus, single point-of-care PVR measurements may overestimate the true prevalence of an abnormal residual urine volume, particularly when higher cutoffs (e.g., PVR ≥ 100 mL) are applied. Differences in maximum daytime PVR and mean 24-h PVR are most likely a function of the number of measurements, as significant interindividual in PVR has been previously reported [20]. Indeed, this is consistent with data from Saaby and colleagues on the repeatability of PVR ≥ 100 mL among women with uro-gynecological complaints, wherein the prevalence of PVR ≥ 100 was 14%, but declined to 1.3% on repeated measurements [25].

The present post hoc study design is limited by the absence of a prospective power analysis, lack of a concomitant control group and small sample size. Moreover, the convenience sample of patients recruited from a tertiary referral hospital might introduce selection biases in this study and limits the generalizability of this study beyond older women with urinary incontinence. Data about urinary tract infection and stage of prolapse are missing and may be confounding factors that may influence the PVR. Measurement errors cannot be excluded, given that a team of nurses was responsible for the PVR measurements, and interrater reliability was not examined. 

In addition, this study relied on 24-h FVCs, which are considered to be less reliable than FVCs of longer duration [26]. Given that there are currently no patient-centered equipment options for home measurement of PVR, the present study design necessitated hospitalization, which is not a viable option in the real-world evaluation of urinary incontinence amongst community-dwelling adults. Future studies should aim to validate our results using three-day FVC_PVR_ instruments and establish precise cut-off values for clinically significant PVR elevations.

Overall, the present study suggests that bladder emptying is comparable in efficacy during the nighttime versus daytime. Moreover, there is no evidence to suggest that measurements of PVR may differentiate the clinical diagnoses of SUI, UUI and MUI in older women. Multiple PVR measurements may be needed to increase intrasubject reliability

## 5. Conclusions

The present analysis did not identify significant circadian variation in mean PVR or Void% among older women with stress, urge or mixed urinary incontinence. This study is the first to utilize the 24-h FVC with PVR volume to characterize the circadian pattern of PVR and Void% in older women with SUI, UUI and MUI, which may have bearing on the rational use and interpretation of one-time point-of-care PVR testing

## Figures and Tables

**Table 1:** Patient characteristics.

Variable	All Patients (*n* = 90)	SUI (*n* = 28)	UUI (*n* = 36)	MUI (*n* = 26)	*p*-Value
Age (years)	76 (74–78)	74 (71–78)	77 (73–79)	78 (74–80)	0.345
BMI (kg/m^2^)	28 (27–29)	28 (26–32)	28 (25–31)	28 (26–29)	0.872
**Gynecological history**					
Parity	2 (2–3)	2 (1–3)	3 (2–3)	2 (1–3)	0.228
vaginal deliveries	2 (2–2)	2 (1–2)	2 (2–3)	1 (1–2)	0.186
cesarean deliveries	0 (0–0)	0 (0–0)	0 (0–0)	0 (0–0)	0.382
Hysterectomy	33 (37%)	16 (57%)	10 (28%)	7 (27%)	0.031 *
Prolapse	68 (76%)	22 (79%)	25 (70%)	21 (81%)	0.583
**Comorbid conditions**					
Chronic kidney disease	14 (15%)	1 (4%)	8 (22%)	5 (19%)	0.090
*Stage 3*	12 (13%)	1 (4%)	7 (19%)	4 (15%)	
*Stage 4*	1 (1%)	0	1 (3%)	0	
*Stage 5*	1 (1%)	0	0	1 (4%)	
Diabetes mellitus	18 (20%)	8 (29%)	9 (25%)	1 (4%)	0.051
Chronic obstructive pulmonary disease	9 (10%)	4 (14%)	3 (8%)	2 (8%)	0.744
Congestive heart failure	9 (10%)	3 (11%)	4 (11%)	2 (8%)	0.908
Lower extremity venous insufficiency	40 (44%)	11 (39%)	18 (50%)	11(42%)	0.687
Sleep apnoea	5 (6%)	2 (7%)	3 (8%)	0	0.371
Severe constipation	21 (23%)	7 (25%)	9 (25%)	5 (20%)	0.859
Depression	15 (17%)	1 (4%)	9 (25%)	5 (20%)	0.048 *
Neurological Disorders	18 (20%)	1 (4%)	11 (31%)	6 (23%)	0.023 *
*Stroke*	7 (8%)	0	7 (20%)	0	
*Parkinson’s disease*	4 (4%)	0	2 (6%)	2 (8%)	
*Normal pressure hydrocephalus*	1 (1%)	0	0	1 (4%)	
*Dementia*	2 (2%)	0	1 (3%)	1 (4%)	
*Inflammatory disease of CNS*	1 (1%)	0	1 (3%)	0	
*Peripheral neuropathy due to iatrogenic lesions*	1 (1%)	1 (4%)	0	0	
*Postpolio syndrome*	1 (1%)	0	0	1 (4%)	
*Spinal canal stenosis*	3 (3%)	0	2 (6%)	1 (4%)	
**Medications**					
Total medications	6 (5–7)	7 (5–8)	6 (4–7)	5 (3–7)	0.460
α-adrenergic agonists	8 (9%)	4 (14%)	3 (8%)	1 (4%)	0.475
α-adrenergic antagonists	0	0	0	0	-
Angiotensin converting enzyme inhibitors	9 (10%)	4 (14%)	2 (6%)	3 (12%)	0.553
Anticholinergics	5 (6%)	1 (4%)	3 (8%)	1 (4%)	0.729
Calcium channel blockers	17 (19%)	6 (21%)	4 (11%)	7 (27%)	0.268
Cholinesterase inhibitors	3 (3%)	0	3 (8%)	0	0.111
Diuretics	18 (20%)	4 (14%)	9 (25%)	5 (19%)	0.601
*Loop diuretics*	7 (8%)	1 (4%)	4 (11%)	2 (8%)	
*Thiazide diuretics*	2 (2%)	0	0	2 (8%)	
*Potassium-sparing diuretics*	3 (3%)	1 (4%)	1 (3%)	1 (4%)	
*Carbonic anhydrase inhibitors*	0	0	0	0	
*Combination: loop and potassium sparing diuretics*	2 (2%)	1 (4%)	1 (3%)	0	
*Combination: thiazide and potassium sparing diuretics*	4 (4%)	1 (4%)	3 (8%)	0	
Opioids	14 (16%)	5 (18%)	6 (17%)	3 (12%)	0.817
Sedatives/hypnotics	16 (18%)	5 (18%)	7 (19%)	4 (15%)	0.941
Antipsychotics	3 (3%)	1 (4%)	2 (6%)	0	0.777
H1 blockers	10 (11%)	5 (18%)	3 (8%)	2 (8%)	0.481
Selective serotonin reuptake inhibitors	16 (18%)	1 (4%)	10 (28%)	5 (20%)	0.032 *
Sodium-glucose cotransporter 2 inhibitors	0	0	0	0	-

Note: Continuous variables are reported as median (95% confidence interval) and categorical variables are reported as frequency (percent). One value was missing for BMI. Chronic kidney disease (CKD), stage is consistent with KDIGO 2012 Clinical Practice Guideline for the evaluation and management of CKD [23]. Abbreviations: SUI—stress urinary incontinence; UUI—urge urinary incontinence; MUI—mixed urinary incontinence; BMI—body mass index; CNS—central nervous system. * Denotes statistical significance.

**Table 2:** Functional status, frailty, risk factors for falling and LUTS-related quality of life.

Variable	All Patients (*n* = 90)	SUI (*n* = 28)	UUI (*n* = 36)	MUI (*n* = 26)	*p*-Value
**Independence in ADLs** (Katz Index) Total score (0–6)					<0.001 *
Severe Impairment (0–2)	13 (14%)	0 (0%)	11 (31%)	2 (8%)	
Moderate Impairment (3–4)	18 (20%)	2 (7%)	8 (22%)	8 (31%)	
Well-preserved function (5–6)	59 (66%)	26 (93%)	17 (47%)	16 (62%)	
**Frailty (TFI)** Total score (0–15)					0.017 *
Nonfrail (0–4)	26 (30%)	13 (48%)	5 (15%)	8 (32%)	
Frail (5–15)	60 (70%)	14 (52%)	29 (85%)	17 (68%)	
**Fall risk factors** (STRATIFY) Total score (0–5)					0.201
Low/moderate risk (0–1)	51 (57%)	20 (71%)	18 (50%)	13 (52%)	
High risk (2–5)	38 (43%)	8 (29%)	18 (50%)	12 (48%)	
**Lower urinary tract symptoms** (ICIQ-fLUTS)					
Total score—Filling (0–16)	6 (6–7)	6 (5–6)	7 (6–7)	7 (5–9)	0.167
Total score—Voiding (0–12)	1 (0–2)	1 (0–2)	2 (0–4)	1 (0–2)	0.346
Total score—Incontinence (0–20)	11 (10–12)	11 (10–12)	11 (8–13)	11 (9–14)	0.823

Note: Continuous variables are reported as median (95% confidence interval) and categorical variables as frequency (percent). Questionnaire data were missing for 4 patients for TFI; 1 patient for STRATIFY; and 1 patient for ICIQ-fLUTS. Abbreviations: SUI—stress urinary incontinence; UUI—urge urinary incontinence; MUI — mixed urinary incontinence; ADL- activities of daily living; TFI—Tilburg Frailty Indicator; STRATIFY—St. Thomas’s Risk Assessment Tool in Falling Elderly Inpatients; ICIQ-fLUTS—International Consultation on Incontinence modular Questionnaire for LUTS in women. (*) Denotes statistical significance.

**Table 3:** FVC_PVR_ characteristics.

	All Patients (*n* = 90)	SUI (*n* = 28)	UUI (*n* = 36)	MUI (*n* = 26)	*p*-Value
24-h volume (mL)	2086 (1827–2260)	2549 (2120–2852)	1747 (1430–2000)	2173 (1827–2523)	<0.001 *
Global polyuria (24-h urine output > 40 mL/kg)	18 (20%)	8 (29%)	2 (6%)	8 (31%)	0.013 *
Nocturia (≥2 voids/night)	60 (67%)	19 (68%)	22 (61%)	19 (73%)	0.586
Nocturnal polyuria (NPi > 33%)	63 (70%)	17 (85%)	25 (74%)	12 (67%)	0.294
Number of voids (24 h)	9 (8–9)	9 (8–10)	8 (7–9)	10 (9–12)	0.005 *
Mean VV (mL)	200 (180–241)	291 (233–325)	177 (157–200)	181 (151–245)	<0.001 *
Max VV (mL)	380 (340–400)	470 (400–500)	335 (225–400)	320 (250–400)	<0.001 *
Total UI weight (g)	45 (19–90)	15 (0–46)	88 (14–265)	48 (22–236)	0.040 *
Mean 24-h PVR (mL)	26 (20–36)	28 (16–39)	27 (10–43)	24 (11–45)	0.837
Mean daytime PVR (mL)	20 (11–30)	22 (10–44)	17 (7–32)	22 (4–37)	0.658
Mean nighttime PVR (mL)	20 (12–42)	20 (9–68)	16 (3–44)	31 (10–55)	0.511
Max 24-h PVR (mL)	92 (67–135)	105 53–156)	77 (40–126)	115 (63–191)	0.463
Mean 24-h Void% (%)	90 (87–90)	91 (87–94)	89 (80–95)	91 (85–95)	0.729
Mean daytime Void% (%)	91 (88–94)	91 (85–96)	91 (82–96)	92 (84–98)	0.876
Mean nighttime Void% (%)	91 (87–96)	94 (84–98)	92 (83–98)	91 (84–98)	0.884
Min 24-h Void% (%)	64 (56–72)	68 (58–75)	59 (49–81)	64 (41–83)	0.555

Note: Continuous variables are reported as median (95% confidence interval) and categorical variables as frequency (percent). Abbreviations: FVC_PVR_—frequency-volume chart with postvoid residual volume; SUI—stress urinary incontinence; UUI—urge urinary incontinence; MUI—mixed urinary incontinence; NPi—nocturnal polyuria index = proportion of nocturnal urine volume of the total 24-h urine volume in the absence of global polyuria; VV—voided volume; UI—urinary incontinence; PVR—postvoid residual; Void%—voided percentage = (VV/[VV + PVR]) × 100%). (*) Denotes statistical significance.

**Table 4:** Distribution of mean 24-h and maximum daytime PVR.

	All Patients (*n* = 90)	SUI (*n* = 28)	UUI (*n* = 36)	MUI (*n* = 26)	*p*-Value
**Mean 24-h PVR**					0.781
<50 mL	66 (73%)	20 (71%)	27 (75%)	19 (73%)	
50–100 mL	18 (20%)	7 (25%)	7 (19%)	4 (15%)	
>100 mL	6 (7%)	1 (4%)	2 (6%)	3 (12%)	
**Max daytime PVR**					0.499
<50 mL	39 (43%)	11 (39%)	16 (44%)	12 (46%)	
50–100 mL	21 (23%)	8 (29%)	10 (28%)	3 (12%)	
>100 mL	30 (33%)	9 (32%)	10 (28%)	11 (42%)	

Note: Variables are reported as frequency (percent). Abbreviations: SUI—stress urinary incontinence; UUI—urge urinary incontinence; MUI—mixed urinary incontinence; PVR—postvoid residual. * Denotes statistical significance.
